# Effect of alloying Li on lithium-ion batteries applicability of two-dimensional TiN and TiC as novel electrode materials: a first principle study

**DOI:** 10.1038/s41598-023-42954-w

**Published:** 2023-09-21

**Authors:** Fatemeh Shirvani, Mohammad Reza Jafari, Aliasghar Shokri

**Affiliations:** 1 Department of Condensed Matter Physics, Faculty of Physics, Alzahra University, Tehran, Iran; 2 Department of Theoretical Physics and Nano, Faculty of Physics, Alzahra University, Tehran, Iran

**Keywords:** Physics, Condensed-matter physics, Surfaces, interfaces and thin films

## Abstract

The two-dimensional structures of transition metal nitride and carbide, TiN, and TiC have been alloyed with lithium (Li) in replacement of Ti, and their Li-ion applicability has been investigated using density functional theory and general gradient approximation. The alloy composition of $x=0.125$, 0.25, 0.375, and 0.5 have been considered and the stability of the alloys has been proved by cohesive energy and phonon density of states results. Moreover, the bond lengths between atoms as structural properties have been studied for these alloy structures. The largest peak of quantum capacitance and the largest negative value of surface storage charge are for alloy composition of TiC with $x=0.125$ with the values of 909.79 $\upmu$F/cm$^{2}$ and $-\,1819.58\,\upmu$C/cm$^{2}$, respectively. Moreover, the results of the quantum capacitance and surface storage charge as a function of voltage for all Li alloy compounds are in the range of excellent supercapacitors and could have good potential to use as an electrode in the capacitor of Li-ion batteries. Furthermore, the electronic density of states of this group of alloys represents metallic behavior and therefore electrode material. In addition, the diffusion coefficient at temperatures of 77 and 300 K has been calculated using molecular dynamic calculations, and its lowest and largest values are $8\times 10^{-8}$ cm$^2$/s (at 77 K) and $5.6\times 10^{-7}$ cm$^2$/s (at 300), respectively. Plus, the largest value of electrical conductivity per relaxation time at 300 K belongs to Li$_{0.25}$Ti$_{0.75}$C with a value of $9.8\times 10^{19}$/($\Omega$ m s).

## Introduction

Transition metal nitrides and carbides are favorable to researchers for many years because of their special properties like resistance to oxidation and corrosion, high hardness, and incompressibility which could make them excellent candidates for many industries like hard coating industries^1–10^. Recently, along with the unique features of this group of compounds, various applications in the field of energy storage have been predicted for them; and due to the benefit of environmentally friendly modality, they could be excellent for clean energy proposes^11–27^. Since these compounds typically represent metallic behavior they could be good choices for electrode materials in energy storage industries. Furthermore, alloying them with elements like magnesium could give them semiconductor properties that can be used as a scatter area for some energy-saving applications like the thermoelectric, supercapacitor, Li-ion batteries, and solar energy industries^8,12,14,15^.

Recently, Zhang et al.^3^ worked on Cellulose nanofibrils/AlN experimentally and reached a high thermal conductivity of more than 4.2 W/m.K for managing the heat in energy storage devices.

Moreover, Gao et al.^28^ studied the effect of alloying Ni on supercapacitance properties of CrN using the arc ion plating method and they got accessed to the value of 58.5 mF/cm$^2$ for the specific capacitance at 1 mA/cm$^2$ for 54.2% presence of Ni and H$_2$SO$_4$ was used as an electrolyte material; and this value was 80 times higher than for the pure CrN.

Wei et al^29^ experimentally worked on VN pillared with Al atoms for the purpose of sodium-ion batteries applications and they achieved specific capacitance with the value of 372 mAh/g at 50 mA/g and an energy density of 78.43 Wh/Kg at the power density of 260 W/Kg.

Using the situ polymerization method Zhang et al.^30^ worked on ZrC nanoparticles and could improve the thermal conductivity of it by 225.16%, 4962 W/m K which is good for energy storage applications.

TiN and TiC are other transition metal nitride/carbides which are noticed recently for energy storage applications^25,31,32^. For example, Dong et al.^31^ worked experimentally on MnO$_2$/TiN nanotubes and they obtained the specific capacitance of 681 F/g at 2 A/g which is suitable for electrode material of supercapacitors. Furthermore, Irfan Ali et al.^32^ studied Mo$_2$TiC$_2$ alloyed with Sn$^{2+}$ ion using the cetyltrimethylammonium bromide method and they measured a specific capacitance of 670 F/g which was more than three times larger than for the pure Mo$_2$TiC$_2$.

Li-ion battery application is one of the energy storage properties of TiN and TiC. Kim et al.^33^ worked on Si/TiN nanocomposites with 33.3 Mol Si by using high energy mechanical milling method and they obtained capacity with the value of 300 mAh/g which suggests a good anode for Li-ion batteries. In addition, Balogun et al.^34^ worked on nanoribbons of TiN by hydrothermal method for Li-ion battery applications and they got access to specific capacitance with a value of 288 mAh/g at the current density of 1675 mA/g. Furthermore, Byeon et al.^35^ experimentally studied TiC MXene as a cathode for the coupled Mg$^{2+}$/Li$^{2+}$ batteries and they measured the capacity with the value of about 120 mAh/g. Using the density functional theory (DFT) method Chen et al.^36^ investigated the Li-ion applicability of TiCN MXene; and for Ti$_3$CN and Ti$_3$CNO$_2$ which were saturated with Li atoms they got access to the capacity with the values of 320 and 269 mAh/g, respectively.

In the present work, the two-dimensional structures of TiN and TiC have been considered, and Li atoms have been alloyed in the replacement of Ti atoms with alloy composition of $x = 0.125$, 0.25, 0.375, and 0.5. The calculations have been done using the DFT method and general gradient approximations (GGA). The computational details, structural, phonon, electronic properties (including quantum capacitance, surface storage charge, electronic density of states, and electrical conductivity per relaxation time), and diffusion coefficient will be discussed in the next sections and finally, the results will be concluded.

## Computational details

**Figure 1:**
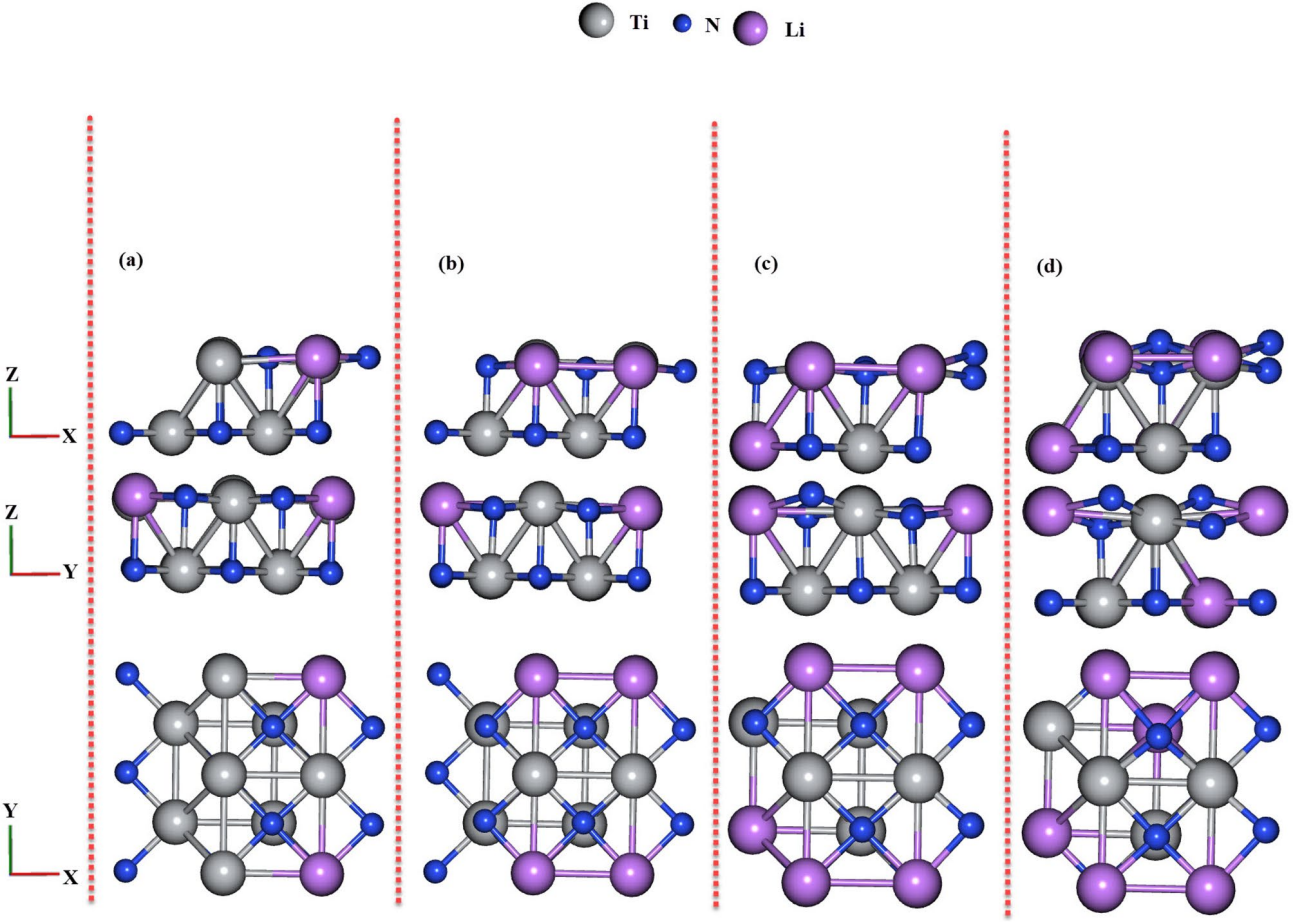
Top view and side views of fully relaxed Li$_x$Ti$_{1-x}$N: (**a**) $x= 0.125$, (**b**) $x= 0.25$, (**c**) $x= 0.375$, and (**d**) $x= 0.5$.

**Figure 2:**
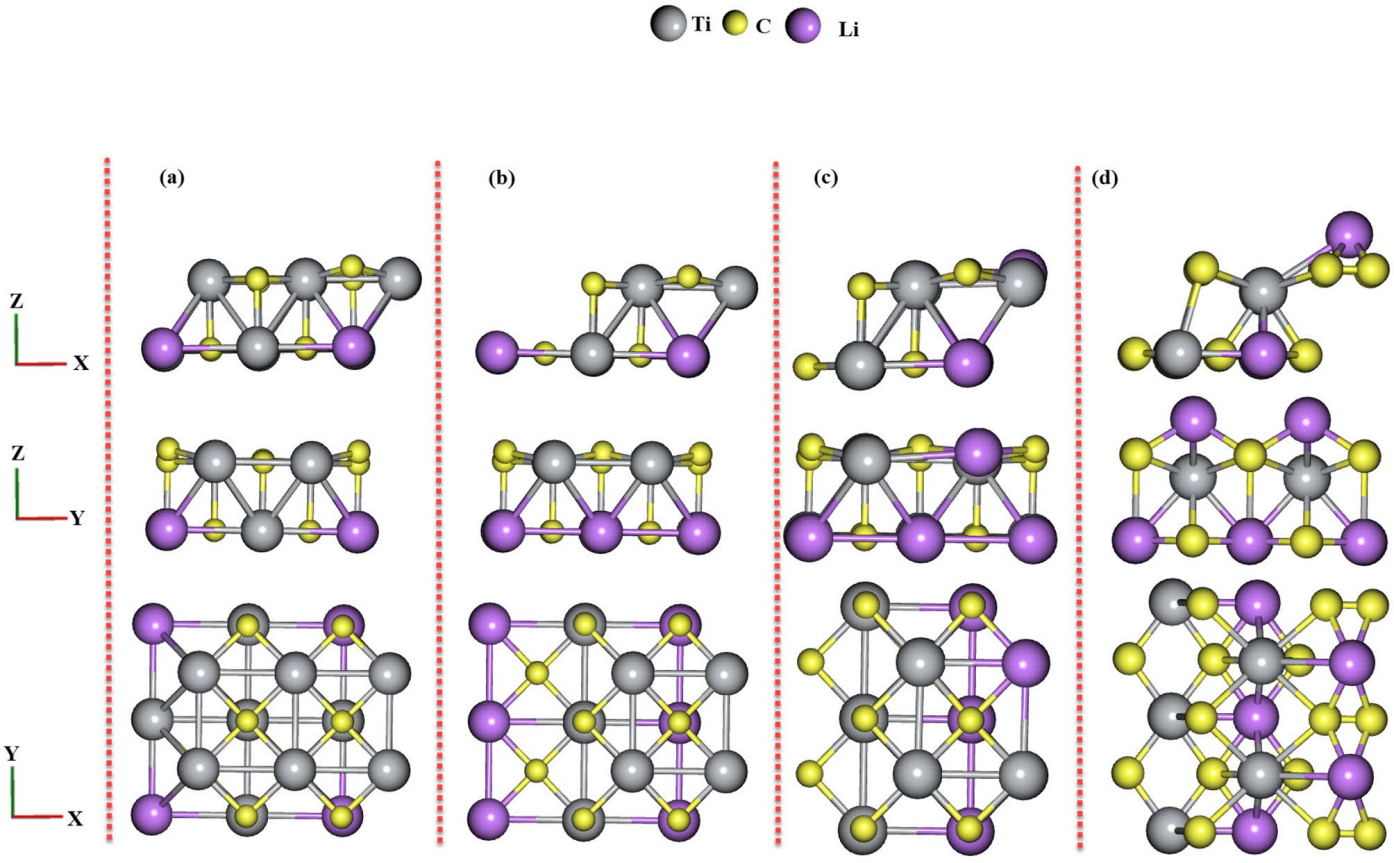
Top view and side views of fully relaxed Li$_x$Ti$_{1-x}$C: (**a**) $x= 0.125$, (**b**) $x= 0.25$, (**c**) $x= 0.375$, and (**d**) $x= 0.5$.

The calculations on this work have been done using the DFT method and GGA approximation with Perdew-Burke-Ernzerhof (PBE)correlation functional and by norm-conserving pseudopotential^37,38^. The slabs have been made at the direction of (110) from the NaCl bulk structure of TiN and TiC. Then the two-dimensional structures of TiN and TiC alloyed with Li (see Figs. 1a–d and 2a–d) have been studied using the Quantum ESPRESSO package^39^. The optimized energy cutoff and k-point mesh were 46 Ry and $10\times 10\times 1$, respectively (these are the points from which the total energy of the structure tends to a constant value). In addition, the structural parameter and atomic position have been optimized and relaxed using the force and energy minimizing method in the order of 0.01 eV/Å and 10$^{-5}$ eV, respectively. The site occupancy factor of each atom has been considered to be 1 and the position of Li atoms for each alloy composition has been chosen according to the lowest energy of each atomic array by using relaxation calculation (the atomic arrays which had the lowest energy were considered as the final Li composition). Moreover, a vacuum of about 27 Å in the z-direction has been considered to avoid the interaction between atoms. Furthermore, to study the phonon properties the density functional perturbation theory (DFPT) has been used^40^.

To calculate the quantum capacitance and surface storage charge the relations $e^2 \int _{- \infty }^{+ \infty } \mathrm{n(E)F}_{\mathrm{F.D}}(E-e\phi _{\textrm{L}})dE$ and $\int _{0}^{ \phi _{\textrm{L}}} \mathrm C_{\textrm{Q}} d\phi$ have been used which e, n, E, F, $\phi _{\textrm{L}}$, and C$_{\textrm{Q}}$ are charge of the electron, the electron density of states, the difference between the energy of the electrons and Fermi level energy, Fermi-Dirac distribution function, local potential, and quantum capacitance, respectively^41–44^.

In addition, the electrical conductivity per relaxation time ($\sigma$/$\tau$) has been investigated using the semiclassical Boltzmann theory and by implementing the BolTzraP package^45–50^.

The calculations have also been done using the GGA+ Hubbard potential (GGA+U) method but no special difference from the GGA results has been achieved, therefore, its results are omitted in this work.

In the next section, first, the structural properties and mechanical stability of the alloy compounds will be discussed, then the phonon properties using the phonon density of states will be studied, and finally, quantum capacitance, surface storage charge, density of state, diffusion coefficient, and electrical conductivity per relaxation time for TiN and TiC in the presence of Li (with *x* = 0.125, 0.25, 0.375, 0.5) will be explained.

## Results and discussions

The top view (XY), and side views (XZ, and YZ) of the relaxed ordered structure of two-dimensional TiN and TiC alloyed with Li atoms with the alloy composition of with $x = 0.125$, 0.25, 0.375, 0.5 have been represented in Figs. 1a–d and 2a–d. According to Fig. 1a–d for TiN alloyed with Li, between the alloy composition of $x = 0.25$ (Fig. 1b) and $x = 0.125$ (Fig. 1a) there is a difference in the position of N atoms (see the top view in the XZ direction) which is related to the bond length between N and Li or Ti atoms. For $x = 0.375$ and $x = 0.5$ (see Fig. 1c–d), there are octahedral distortions because of the changes in bond length between N atoms and Ti or Li atoms in comparison with $x = 0.25$ which are obvious in top and side views. In addition, for $x = 0.5$ in comparison with $x = 0.375$, there are changes in the placement of N atoms which can be seen in the side views of the structures.

According to the Fig. 2a–b, for TiC alloyed with Li atoms there is an obvious octahedral distortion in the arrays of C atoms for $x = 0.25$ in comparison with $x = 0.125$ which can be seen in the side view in the XZ direction. Furthermore, for $x = 0.375$ (see Fig. 2c), there are differences in the placement of C atoms in comparison with $x = 0.25$ and it can be seen in the top view and side (in the XZ direction) views, while for $x = 0.5$ (see Fig. 2d) the replacement of C atoms in comparison with $x = 0.375$ is more visible and can be seen in top view and both side views.

**Figure 3:**
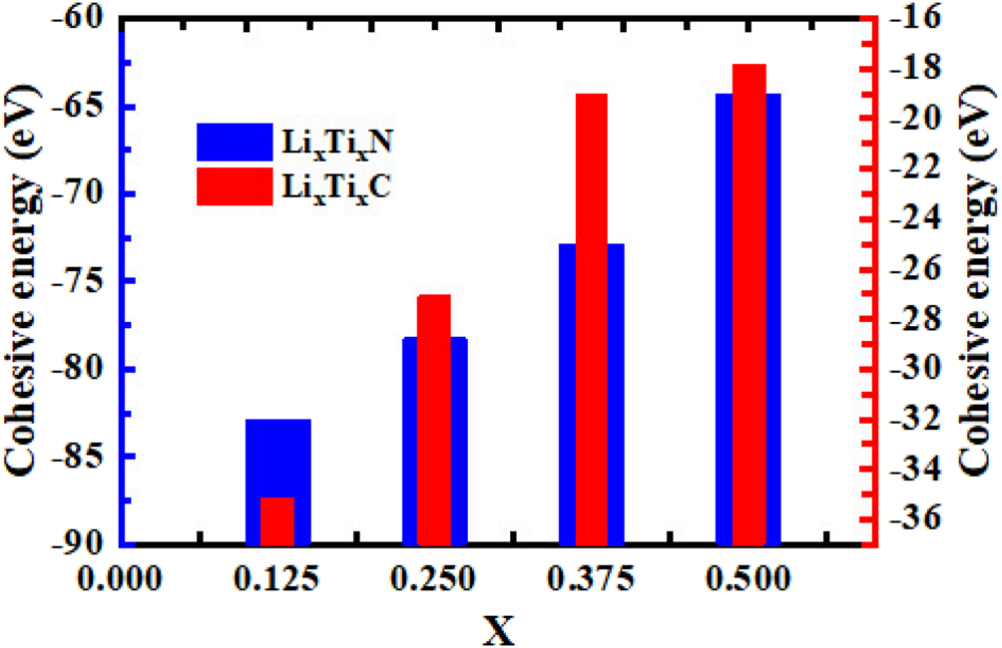
Cohesive energy as a function of alloy composition ($x = 0.125$, 0.25, 0.375, and 0.5) for Li$_x$Ti$_{1-x}$N and Li$_x$Ti$_{1-x}$C.

To evaluate the structural and mechanical stability of the alloy compounds the cohesive energies as a function of alloy composition have been calculated and represented in Fig. 3 for Li alloys of TiN (blue bar graph) and TiC (red bar graph). The cohesive energy is calculated using the following formula^51^:

1 $$\begin{aligned} \textrm{E}_{\textrm{Coh}}=\mathrm E_{\textrm{tot}} - \sum \mathrm{m_lE}_{\mathrm{x_l}} \end{aligned}$$

Here, E$_{\textrm{tot}}$, m, and E$_{\mathrm{x_l}}$ are related to the total energy of the alloys, number of each element (Li, Ti, N or C) in the alloy compounds, and the total energy just for one atom of each kind of element, respectively.

According to Fig. 3, the cohesive energy for all of Li$_x$Ti$_{1-x}$N and Li$_x$Ti$_{1-x}$C is negative which refers to the structural and mechanical stability of these alloys compounds. The largest negative value for Li$_x$Ti$_{1-x}$N alloys belongs to $x = 0.125$ with a value of − 82.86 eV and the lowest negative value is related to $x = 0.5$ with a value of − 64.29 eV. Moreover, the largest negative value of cohesive energy for Li$_x$Ti$_{1-x}$C is for $x = 0.125$ with a value of − 35.13 eV while its lowest negative value is − 17.85 eV for $x = 0.5$. In addition, the cohesive energy values for alloys of TiN have larger negative values in comparison with TiC alloy which indicates more structural and mechanical stability of TiN alloys.

**Figure 4:**
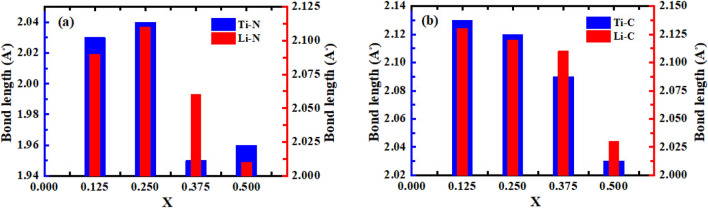
Bond lengths (Ti–N, Li–N, Ti–C, and Li–C) as a function of alloy composition ($x = 0.125$, 0.25, 0.375, and 0.5) for (**a**) Li$_x$Ti$_{1-x}$N, and (**b**) Li$_x$Ti$_{1-x}$C.

The bond length between atoms as a function of alloy composition has been depicted in Fig. 4a–b and the blue bar graphs are related to the bond length of Ti with N or C atoms while the red bar graphs are related to the bond lengths of Li atoms with N or C atoms. The larger value of bond length refers to the weaker bond between atoms and the lower values refer to the more powerful bonds. According to Fig. 4a, the largest bond length between Ti and N atoms belongs to $x = 0.25$ with a value of 2.04 Å  while the lowest value of Ti–N is 1.95 Å and is related to $x = 0.375$. Furthermore, the largest value for bond length between Li and N atoms belongs to $x = 0.25$ and equals 2.11 Å while the lowest Li–N is related to $x = 0.5$ with a value of 2.01 Å. As can be seen in Fig. 4b, the highest values of Ti–C and Li–C belong to $x = 0.125$ with a value of 2.13 Å  and the lowest values of them are related to $x = 0.5$ with a value of 2.03 Å. In addition, for the $x = 0.375$, the Ti–C and Li–C are 2.09 and 2.11 Å  respectively; while for $x = 0.25$, both Ti–C and Li–C have the same values of 2.12 Å.

**Table 1:** The calculated enthalpy of formation energy (kJ/mol) for Li$_x$Ti$_{1-x}$N and Li$_x$Ti$_{1-x}$C.

Compounds	Enthalpy of formation energy (kJ/mol)
Li$_{0.125}$Ti$_{0.875}$N	$-$ 7954.56
Li$_{0.25}$Ti$_{0.75}$N	$-$ 7513.92
Li$_{0.3755}$Ti$_{0.625}$N	$-$ 6996.48
Li$_{0.5}$Ti$_{0.5}$N	$-$ 6171.84
Li$_{0.125}$Ti$_{0.875}$C	$-$ 3372.48
Li$_{0.25}$Ti$_{0.75}$C	$-$ 2599.68
Li$_{0.375}$Ti$_{0.625}$C	$-$ 1824.96
Li$_{0.5}$Ti$_{0.5}$C	$-$ 1713.6

The thermal stability of the alloy compositions has been investigated by calculating the enthalpy of formation energy. According to Table 1, the large negative values of enthalpy of formation energy for all alloy composition refers to the thermal stability of the alloys.

**Figure 5:**
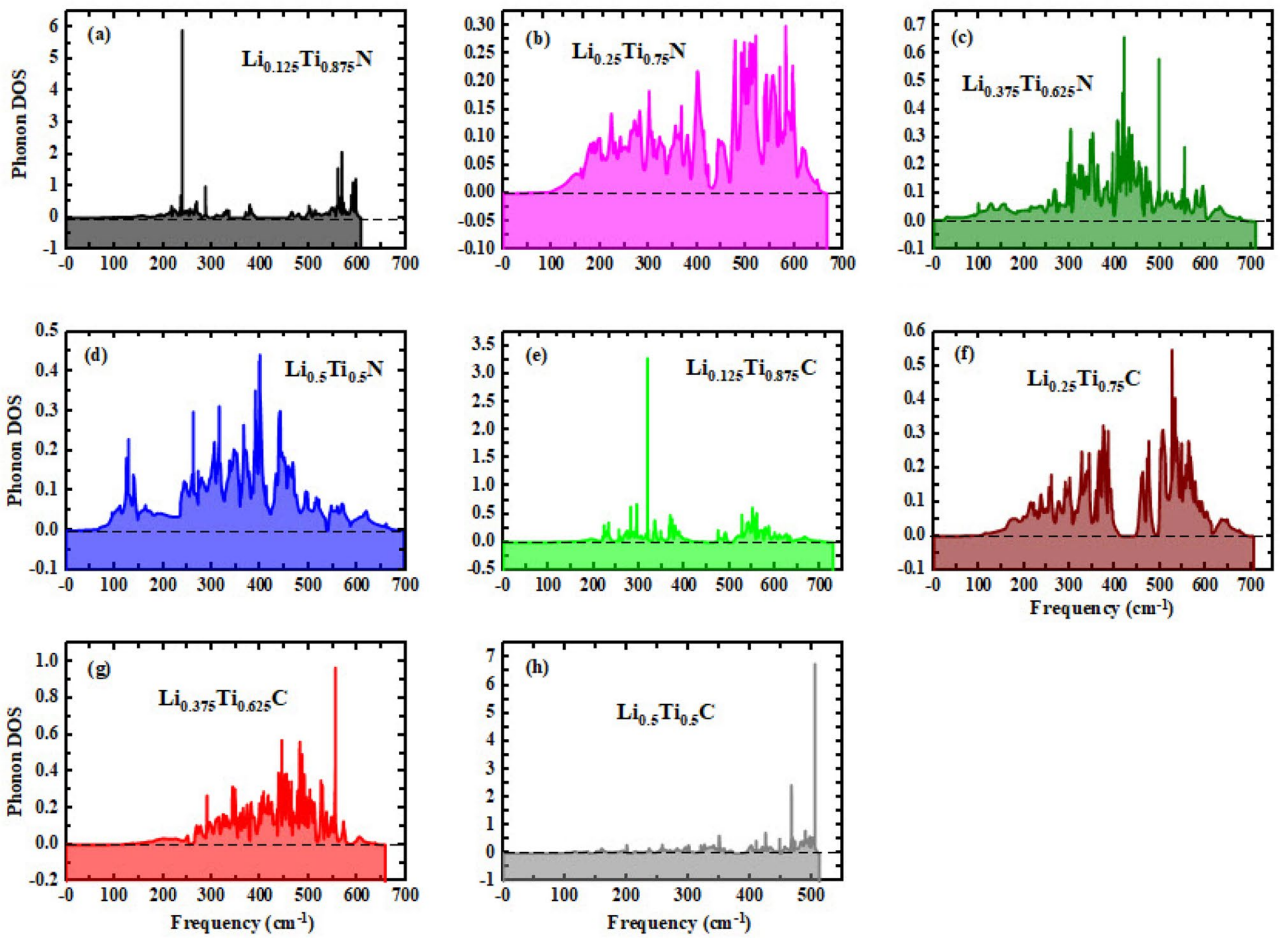
Phonon density of states (Phonon DOS) as a function of frequency for (**a**) Li$_{0.125}$Ti$_{0.875}$N, (**b**) Li$_{0.25}$Ti$_{0.75}$N, (**c**) Li$_{0.375}$Ti$_{0.625}$N, (**d**) Li$_{0.5}$Ti$_{0.5}$N, (**e**) Li$_{0.125}$Ti$_{0.875}$C, (**f**) Li$_{0.25}$Ti$_{0.75}$C, (**g**) Li$_{0.375}$Ti$_{0.625}$C, and (**h**) Li$_{0.5}$Ti$_{0.5}$C (the zero point has been shown by dash lines).

The phonon density of states (phonon DOS) as a function of frequency has been calculated for Li alloys of both TiN and TiC and has been depicted in Fig. 5a–h. Positive modes and zero values of phonon DOS at zero frequency for all alloys indicate the dynamic stability of these alloy compounds. According to Fig. 5e–d, the largest peak for TiN with the alloy composition of $x = 0.125$ is 5.91 and occurs at the frequency of 239.04 cm$^{-1}$, while for $x = 0.25$, 0.375, and 0.5 are 0.3, 0.12, and 0.42 at 581.89, 435.08, and 397.94 cm$^{-1}$, respectively. In addition, according to Fig. 5e–h, for TiC alloys the highest peak for alloy composition of $x = 0.125$, 0.25, 0.375, and 0.5 are 3.28, 0.55, 0.56, and 6.77 at the frequencies of 318.62, 526.18, 444.33, and 504.77 cm$^{-1}$, respectively.

Furthermore, for TiN with Li composition of $x = 0.125$, there are two gaps between modes with the values of 28.01 cm$^{-1}$ (between 336.38 and 364.39 cm$^{-1}$), and 60.29 cm$^{-1}$ (between 395.38 and 455.67 cm$^{-1}$). Moreover, for TiC with alloy composition of $x = 0.125$, there is a gap with a value of 6.87 cm$^{-1}$ (between 498.07 and 504.94 cm$^{-1}$ ), however, the values of phonon DOS at upper than 400 cm$^{-1}$ until 504.94 cm$^{-1}$ are close to zero. For alloy composition of $x = 0.25$ of TiC, there is an obvious gap between modes with a value of 20.56 cm$^{-1}$ (between 420.16 and 440.72 cm$^{-1}$); while for $x = 0.375$, there is a gap with a value of 10.16 cm$^{-1}$ (between 374.16 and 384.32 cm$^{-1}$). All of these gaps could refer to the low phonon contribution of thermal conductivity of the alloys at these modes which could be applicable for saving energy industries like thermoelectric applications (as the phonon contribution of thermal conductivity is the dominant contribution in total thermal conductivity).

**Figure 6:**
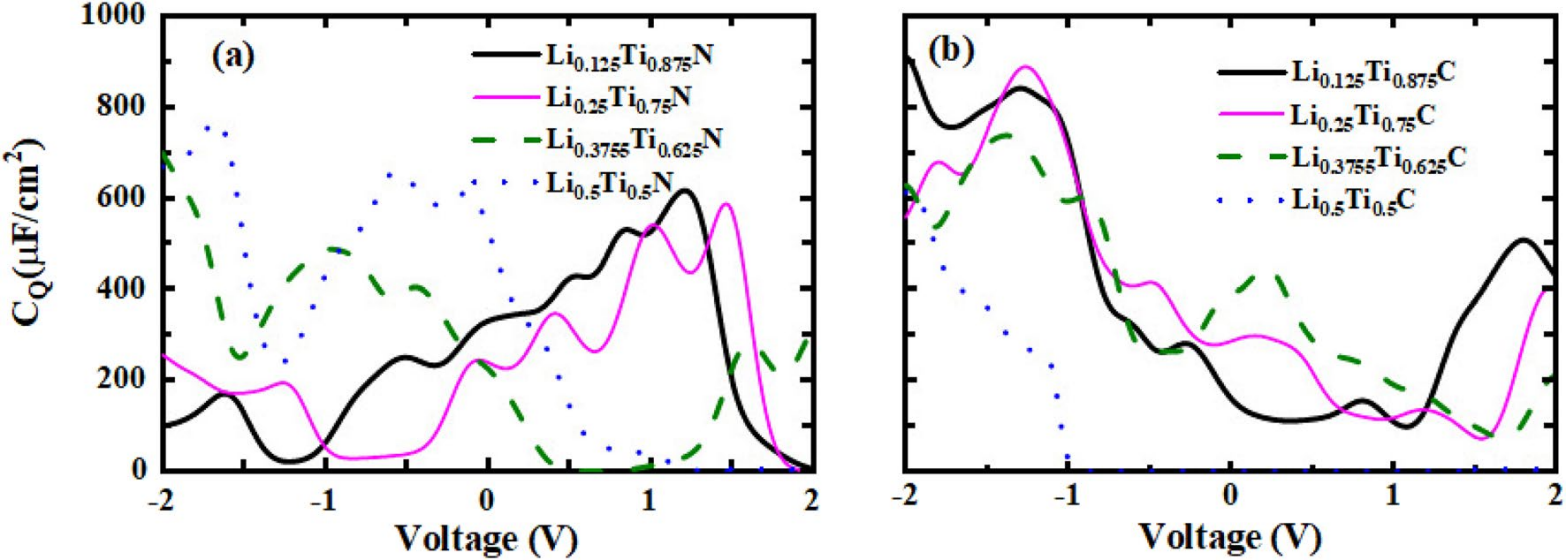
Quantum capacitance as a function of voltage for (**a**) Li$_x$Ti$_{1-x}$N, and (**b**) Li$_x$Ti$_{1-x}$C.

The quantum capacitance (C$_{Q}$) for Li alloys of TiN and TiC as a function of voltage (from − 2 to + 2 V) have been calculated and represented in Fig. 6a and b. According to Fig. 6a, the largest value of C$_{Q}$ for TiN alloy is related to alloy composition of $x = 0.5$ at a negative voltage of − 1.67 V with a value of 772.35 $\upmu$F/cm$^{2}$; and the lowest maximum belongs to alloy composition of $x = 0.25$ with a value of 587.2 $\upmu$F/cm$^{2}$ at the positive voltage of 1.47 V. Moreover, the largest peak for $x = 0.125$ (616.49 $\upmu$F/cm$^{2}$) and $x = 0.375$ (699.34 $\upmu$F/cm$^{2}$) occur at the positive (1.21 V) and negative (− 2 V) voltage, respectively. The largest peak for TiC alloy compositions belongs to $x = 0.125$ at the negative voltage (− 2 V) with a value of 909.79 $\upmu$F/cm$^{2}$, and the lowest maximum is related to $x = 0.5$ with a value of 618.25 $\upmu$F/cm$^{2}$ at − 1.97 V. In addition, the largest peaks of $x = 0.25$ (888.82 $\upmu$F/cm$^{2}$) and $x = 0.375$ (737.1 $\upmu$F/cm$^{2}$) occur at negative voltages of − 1.27, and − 1.38 V, respectively. Moreover, for $x = 0.5$, the values of C$_Q$ tend to zero from − 1 V (the values are in the order of 10$^{-16}$–10$^{-42}$ at the positive voltages).

The results of C$_Q$, at both positive and negative voltages, exhibit large values which are in the range of anode and cathode for supercapacitors, and because of the presence of Li atoms, they could be useful as an electrode in the capacitor of the Li–ion batteries.

Present results are typically in the order of 10 magnitudes larger than the results of Yang et al.^52^ for graphene-based materials. In addition, in comparison with Wang et al.^53^ results for graphene doped with metal and codoped with nitrogen and metal, the present results of quantum capacitance are typically 5 times larger.

**Figure 7:**
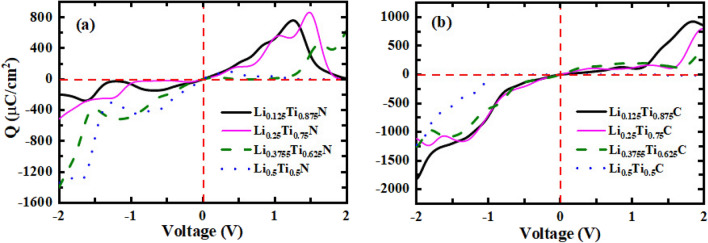
Surface storage charge as a function of voltage for (**a**) Li$_x$Ti$_{1-x}$N, and (**b**) Li$_x$Ti$_{1-x}$C.

The surface storage charge (Q) has been calculated for both TiN and TiC alloys and depicted in Fig. 7a–b. According to Fig. 7a, the largest negative value of TiN alloys at negative voltages is related to the alloy composition of $x = 0.375$ at − 2 V with a value of − 1398.68 $\upmu$C/cm$^{2}$, and at positive voltages, the largest peak occurs for $x = 0.25$ at 1.48 V with a value of 865.26 $\upmu$C/cm$^{2}$. Furthermore, according to Fig. 7b, the largest negative value of Q for TiC alloy compositions belongs to $x = 0.125$ at − 2 V with a value of − 1819.58 $\upmu$C/cm$^{2}$, and at positive voltages, the largest peak also belongs to this alloy composition with a value of 924.63 $\upmu$C/cm$^{2}$ at 1.84 V.

The present results for Q are compatible with the results of the others which introduce the materials with supercapacitance performance^44,54,55^. For example, in comparison with Rani et al.^54^ work for Tellurene bilayer derivatives, the present results for Li alloy of TiN and TiC are larger.

**Figure 8:**
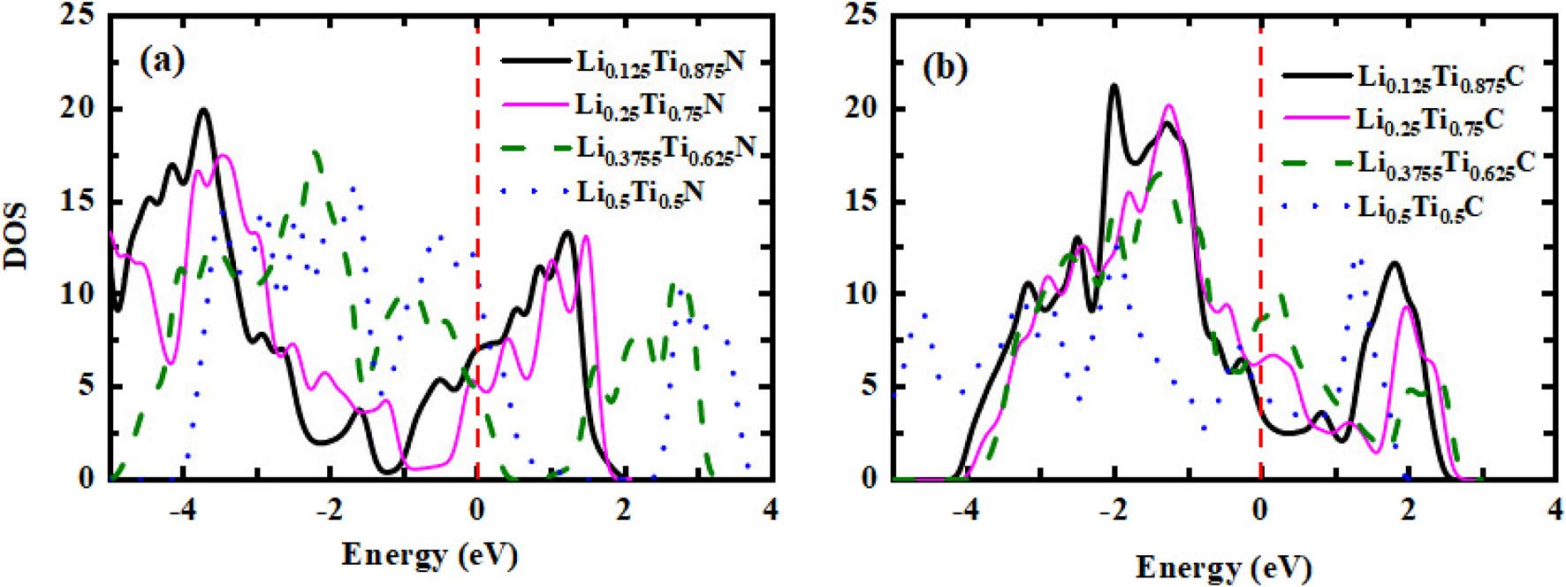
Electronic density of states (DOS) as a function of energy for (**a**) Li$_x$Ti$_{1-x}$N, and (**b**) Li$_x$Ti$_{1-x}$C.

The electronic density of states as a function of energy for Li alloy of TiN and TiC have been calculated using k-point mesh of $25\times 25\times 1$ and have been presented in Fig. 8a and b by setting the Fermi level at zero point. As can be seen for all of the alloy compositions of both TiN and TiC the Fermi level is crossed by the curves which refers to the metallic behavior of these alloys. Moreover, for TiN alloys the largest peak at valence and conduction bands belongs to $x = 0.125$ at − 3.73 eV with a value of 19.96 and at 1.23 eV with a value of 13.35. In addition, for TiC alloy the highest peak at the valence band is related to $x = 0.125$ with a value of 21.27 at − 2.01 eV; while for the conduction band, the largest peak belongs to $x = 0.5$ with a value of 12.45 at 1.3 eV.

The diffusion coefficient for Li$_x$Ti$_{1-x}$N and Li$_x$Ti$_{1-x}$C at temperatures of 77 and 300 K (which are important temperatures for transport properties) has been calculated using molecular dynamic (MD) calculations and collected in Table 2. According to Table 2, the lowest and largest value of the diffusion coefficient at 77 K have values of $8\times 10^{-8}$ cm$^2$/s (belongs to Li$_{0.125}$Ti$_{0.875}$N, Li$_{0.125}$Ti$_{0.875}$C, and Li$_{0.25}$Ti$_{0.75}$C) and $1.5\times 10^{-7}$ cm$^2$/s (belongs to Li$_{0.5}$Ti$_{0.5}$C), respectively. Furthermore, at 300 K the lowest and largest values are $2.8\times 10^{-7}$ cm$^2$/s (belongs to Li$_{0.125}$Ti$_{0.875}$C, and Li$_{0.25}$Ti$_{0.75}$C) and $5.6\times 10^{-7}$ cm$^2$/s (belongs to Li$_{0.5}$Ti$_{0.5}$C), respectively.

**Table 2:** The calculated diffusion coefficient (cm$^2$/s) for Li$_x$Ti$_{1-x}$N and Li$_x$Ti$_{1-x}$C at temperatures of 77 and 300 K (D$_{77}$, and D$_{300}$ refer to diffusion coefficient at 77 and 300 K, respectively).

	D$_{77}$ (cm$^2$/s)	D$_{300}$ (cm$^2$/s)
Li$_{0.125}$Ti$_{0.875}$N	$8 \times 10^{-8}$	$4\times 10^{-7}$
Li$_{0.25}$Ti$_{0.75}$N	$1 \times 10^{-7}$	$4.1\times 10^{-7}$
Li$_{0.3755}$Ti$_{0.625}$N	$1.2\times 10^{-7}$	$4.8\times 10^{-7}$
Li$_{0.5}$Ti$_{0.5}$N	$1.2\times 10^{-7}$	$4.4\times 10^{-7}$
Li$_{0.125}$Ti$_{0.875}$C	$8 \times 10^{-8}$	$2.8\times 10^{-7}$
Li$_{0.25}$Ti$_{0.75}$C	$8\times 10^{-8}$	$2.8\times 10^{-7}$
Li$_{0.375}$Ti$_{0.625}$C	$1\times 10^{-7}$	$3.9\times 10^{-7}$
Li$_{0.5}$Ti$_{0.5}$C	$1.5\times 10^{-7}$	$5.6\times 10^{-7}$

The electrical conductivity per relaxation time, $\sigma$/$\tau$ has been calculated at room temperature (300 K) by using the semiclassical Boltzmann theory, and the largest peaks for alloy compositions along with related chemical potential have been collected in Table 3. According to Table 3, all of the largest peaks occur at negative chemical potential and the largest value belongs to Li$_{0.25}$Ti$_{0.75}$C (with a value of $9.8\times 10^{19}$/($\Omega$ m s) and at the chemical potential of − 2.19 eV). Moreover, the lowest value belongs to Li$_{0.5}$Ti$_{0.5}$N (with a value of $3.43\times 10^{19}$/($\Omega$ m s) and at the chemical potential of − 3.88 eV).

**Table 3:** The calculated electrical conductivity per relaxation time, $\sigma$/$\tau$ (1/$\Omega$ m s) for Li$_x$Ti$_{1-x}$N and Li$_x$Ti$_{1-x}$C along with related chemical potential (eV) at room temperature (300 K).

	$\sigma {/}\tau$ (1/$\Omega$ m s)	Chemical potential (eV)
Li$_{0.125}$Ti$_{0.875}$N	$7.62\times 10^{19}$	$-$ 0.33
Li$_{0.25}$Ti$_{0.75}$N	$4.14 \times 10^{19}$	$-$ 4.19
Li$_{0.3755}$Ti$_{0.625}$N	$3.58\times 10^{19}$	$-$ 3.02
Li$_{0.5}$Ti$_{0.5}$N	$3.43\times 10^{19}$	$-$ 3.88
Li$_{0.125}$Ti$_{0.875}$C	$7.37 \times 10^{19}$	$-$ 1.45
Li$_{0.25}$Ti$_{0.75}$C	$9.8\times 10^{19}$	$-$ 2.19
Li$_{0.375}$Ti$_{0.625}$C	$6.27\times 10^{19}$	$-$ 1.47
Li$_{0.5}$Ti$_{0.5}$C	$6.5\times 10^{19}$	$-$ 4.31

## Conclusions

The calculations in this work, have been done with the DFT method and GGA approximation on the two-dimensional structures of TiN and TiC alloyed with Li. The cohesive energy values for all alloy compositions were negative which approved the structural and mechanical stability of them. Moreover, the phonon calculations showed no negative modes for all the alloys of both TiN and TiC which indicated their dynamic stability. In addition, the gap between modes of phonon DOS could refer to low phonon contribution of thermal conductivity which introduced the potential candidate for thermoelectric applications. Furthermore, the peaks of quantum capacitance and the values of surface storage charge at the negative and positive voltage were competitive with the materials which were known as electrode materials for supercapacitors, and so these alloy compositions can be useful as electrodes of capacitors for Li-ion batteries and improve their efficiency. In addition, the electronic density of states of all of the alloy compositions showed metallic behavior. Moreover, using molecular dynamic calculations, the diffusion coefficient at temperatures of 77 and 300 K was calculated, and its lowest and largest values were $8\times 10^{-8}$ cm$^2$/s (at 77 K) and $5.6\times 10^{-7}$ cm$^2$/s (at 300 K), respectively. Finally, the electrical conductivity per relaxation time was studied at room temperature using semiclassical Boltzmann theory, and the largest peak was $9.8 \times 10^{19}$/($\Omega$ m s) and belonged to Li$_{0.25}$Ti$_{0.75}$C.

## Data Availability

The datasets used and/or analysed during the current study available from the corresponding author on reasonable request.

## References

[1] Toth, L. E. *Transition Metal Carbides and Nitrides. Refractory Materials* (Academic Press, 1971).

[2] Oyama, S. T. *The Chemistry of Transition Metal Carbides and Nitrides* (Springer, 1996).

[3] Zhang K, Tao P, Zhang Y, Liao X, Nie S (2019). Highly thermal conductivity of cnf/aln hybrid films for thermal management of flexible energy storage devices. Carbohydr. Polym..

[4] Abedi Ravan B, Jafari H (2019). Dft study on electronic and optical properties of halogen-adsorbed hexagonal boron nitride. Comput. Condens. Matter.

[5] Rostami Osanloo M, Saadat A, van de Put ML, Laturia A, Vandenberghe WG (2021). Transition-metal nitride halide dielectrics for transition-metal dichalcogenide transistors. Nanoscale.

[6] Zeng R, Yang Y, Feng X, Li H, Gibbs LM, DiSalvo FJ, Abruna HD (2022). Nonprecious transition metal nitrides as efficient oxygen reduction electrocatalysts for alkaline fuel cells. Sci. Adv..

[7] Shirvani F, Shokri A, Abedi Ravan B (2021). An ab-initio study of structure and mechanical properties of rocksalt ZrN and its bilayers. Solid State Commun..

[8] Shirvani F, Shokri A, Abedi Ravan B (2023). The role of alloying carbon on thermodynamic properties of ZrN: A first principle study. Solid State Commun..

[9] Shirvani F, Shokri A (2023). Photocatalytic applicability of HfN and tuning it with Mg and Sc alloys: A dft and molecular dynamic survey. Phys. B: Condens. Matter.

[10] Shirvani F, Shokri A, Abedi Ravan B (2021). Electronic, elastic and thermodynamic properties of Ti_x_Zr_1−x_N compounds determined via first-principles calculations. Phys. B Condens. Matter.

[11] Anasori B, Lukatskaya MR, Gogotsi Y (2017). 2d metal carbides and nitrides (mxenes) for energy storage. Nat. Rev. Mater..

[12] Gharavi MA, Armiento R, Alling B, Eklund P (2021). Theoretical study of the phase transitions and electronic structure of (Zr0.5, Mg0.5)N and (Hf0.5, Mg0.5)N. J. Mater. Sci..

[13] Zebarjadi M, Bian Z, Singh R, Shakouri A, Wortman R, Rawat V, Sands T (2009). Thermoelectric transport in a ZrN/ScN superlattice. J. Electron. Mater..

[14] Shirvani F, Shokri A, Abedi Ravan B, Akhoundi Khezrabad MS (2022). Alloying of monolayer zirconium nitride with magnesium and investigating its thermoelectric properties using dft calculations. Solid State Commun..

[15] Shirvani F, Masoudi M (2022). Energy storage applicability of novel two-dimensional transition metal nitride alloys: First principle study. Solid State Commun..

[16] Cheng Y, Yang L, Yin S (2023). Synthesis and lithium ion storage performance of novel two dimensional vanadium niobium carbide (vnbctx) mxene. Compos. Commun..

[17] Wang Di, Zhou C, Filatov AS, Cho W, Lagunas F, Wang M, Vaikuntanathan S, Liu C, Klie RF, Talapin DV (2023). Direct synthesis and chemical vapor deposition of 2d carbide and nitride mxenes. Science.

[18] Dinh K, Liang Q, Du C, Zhao J, Tok A, Mao H, Yan Q (2019). Nanostructured metallic transition metal carbides, nitrides, phosphides, and borides for energy storage and conversion. Nano Today.

[19] Feng K, Li Y, Xu C, Zhang M, Yang X, Cheng Y, Wang Y, Yang L, Yin S (2023). In-situ partial oxidation of tivctx derived TiO_2_ and V_2_O_5_ nanocrystals functionalized tivctx mxene as anode for lithium-ion batteries. Electrochim. Acta.

[20] Lim K, Handoko AD, Nemani S, Wyatt B, Jiang H, Tang J, Anasori B, Seh Z (2020). Rational design of two-dimensional transition metal carbide/nitride (mxene) hybrids and nanocomposites for catalytic energy storage and conversion. ACS Nano.

[21] Liu W, Cao J, Song F, Zhang D, Okhawilai M, Yi J, Qin J, Zhang X (2023). A double transition metal Ti_2_NbC_2_T_x_ mxene for enhanced lithium-ion storage. Rare Met..

[22] Shinde PA, Olabi A, Chodankar NR, Patil SJ, Hwang S, Abdelkareem M (2023). Realizing superior redox kinetics of metal-metal carbides/carbon coordination supported heterointerface for stable solid-state hybrid supercapacitor. Chem. Eng. J..

[23] Sreehari S, George Nithya S, Jose L, Nandakumar S, Subramaniam RT, Aravind A (2023). A review on 2d transition metal nitrides: Structural and morphological impacts on energy storage and photocatalytic applications. J. Alloys Compd..

[24] Mohan S, Revanasiddappa M, Vellakkat M (2023). Conducting polymer/transition metal carbide nanocomposite treated graphite felt as positive electrode in all-vanadium redox flow battery. Synth. Met..

[25] Xiao J, Yu P, Zhao K, Gao H (2023). Two-dimensional transition metal carbide (Ti_0.5_V_0.5_)_3_C_2_T_x_ mxene as high performance electrode for flexible supercapacitor. J. Colloid Interface Sci..

[26] Zhong Y, Xia X, Shi F, Zhan J, Tu J, Fan H (2016). Transition metal carbides and nitrides in energy storage and conversion. Adv. Sci..

[27] Zhang C, Ma Y, Zhang X, Abdolhosseinzadeh S, Sheng H, Lan W, Pakdel A, Heier Ja, Nuesch F (2020). Two-dimensional transition metal carbides and nitrides (mxenes): Synthesis, properties, and electrochemical energy storage applications. Energy Environ. Mater..

[28] Gao Z, Wan Z, Wu Z, Huang X, Li H, Zhang T, Mayrhofer P, Wang Q (2021). Synthesis and electrochemical properties of nanoporous crn thin film electrodes for supercapacitor applications. Mater. Des..

[29] Wei S, Wang C, Chen S, Zhang P, Zhu K, Wu C, Song P, Wen W, Song L (2020). Dial the mechanism switch of vn from conversion to intercalation toward long cycling sodium-ion battery. Adv. Energy Mater..

[30] Zhang Z, Fang J, Mu J (2020). Zrc nanoparticle-modified microencapsulated phase change materials with enhanced thermal conductivity and photo-thermal conversion performance. Int. J. Energy Res..

[31] Dong S, Chen X, Gu L, Zhou X, Li L, Liu Z, Han P, Xu H, Yao J, Wang H, Zhang X, Shang C, Cui G, Chen L (2011). One dimensional mno2/titanium nitride nanotube coaxial arrays for high performance electrochemical capacitive energy storage. Energy Environ. Sci..

[32] Ali I, Haider Z, Rizwan S (2022). Enhanced pseudocapacitive energy storage and thermal stability of Sn^2+^ ion-intercalated molybdenum titanium carbide (mo2tic2) mxene. RSC Adv..

[33] Kim I (1999). Si/tin nanocomposites novel anode materials for li-ion batteries. Electrochem. Solid-State Lett..

[34] Balogun M, Yu M, Li C, Zhai T, Liu Y, Lu X, Tong Y (2014). Facile synthesis of titanium nitride nanowires on carbon fabric for flexible and high-rate lithium ion batteries. J. Mater. Chem..

[35] Byeon A, Zhao M, Ren CE, Halim J, Kota S, Urbankowski P, Anasori B, Barsoum MW, Gogotsi Y (2017). Two-dimensional titanium carbide mxene as a cathode material for hybrid magnesium/lithium-ion batteries. ACS Appl. Mater. Interfaces.

[36] Chen X, Kong Z, Li N, Zhao X, Sun C (2016). Proposing the prospects of ti3cn transition metal carbides (mxenes) as anodes of li-ion batteries: A dft study. Phys. Chem. Chem. Phys..

[37] Perdew JP, Yue W (1986). Accurate and simple density functional for the electronic exchange energy: Generalized gradient approximation. Phys. Rev. B.

[38] Perdew JP, Burke K, Ernzerhof M (1996). Generalized gradient approximation made simple. Phys. Rev. Lett..

[39] Giannozzi P, Baroni S, Bonini N, Calandra M, Car R, Cavazzoni C, Ceresoli D, Chiarotti GL, Cococcioni M, Dabo I, Dal Corso A, de Gironcoli S, Fabris S, Fratesi G, Gebauer R, Gerstmann U, Gougoussis Ch, Kokalj A, Lazzeri M, Martin-Samos L, Marzari N, Mauri F, Mazzarello R, Paolini S, Pasquarello A, Paulatto L, Sbraccia C, Scandolo S, Sclauzero G, Seitsonen AP, Smogunov A, Umari P, Wentzcovitch RM (2009). Quantum espresso: A modular and open-source software project for quantum simulations of materials. J. Condens. Matter Phys..

[40] Giannozzi P, Andreussi O, Brumme T, Bunau O, Buongiorno Nardelli M, Calandra M, Car R, Cavazzoni C, Ceresoli D, Cococcioni M, Colonna N, Carnimeo I, Dal Corso A, de Gironcoli S, Delugas P, DiStasio RA, Ferretti A, Floris A, Fratesi G, Fugallo G, Gebauer R, Gerstmann U, Giustino F, Gorni T, Jia J, Kawamura M, Ko H-Y, Kokalj A, Küçükbenli E, Lazzeri M, Marsili M, Marzari N, Mauri F, Nguyen NL, Nguyen H-V, Otero-de-la Roza A, Paulatto L, Poncé S, Rocca D, Sabatini R, Santra B, Schlipf M, Seitsonen AP, Smogunov A, Timrov I, Thonhauser T, Umari P, Vast N, Wu X, Baroni S (2017). Advanced capabilities for materials modelling with quantum espresso. J. Condens. Matter Phys..

[41] John DL, Castro LC, Pulfrey DL (2004). Quantum capacitance in nanoscale device modeling. J. Appl. Phys..

[42] Hirunsit P, Liangruksa M, Khanchaitit P (2016). Electronic structures and quantum capacitance of monolayer and multilayer graphenes influenced by Al, B, N and P doping, and monovacancy: Theoretical study. Carbon.

[43] Xu Q, Yang G, Fan X, Zheng W (2019). Improving the quantum capacitance of graphene-based supercapacitors by the doping and co-doping: First-principles calculations. ACS Omega.

[44] Zhou Q, Ju W, Yong Y, Zhang Q, Liu Y, Li J (2020). Effect of the n/p/s and transition-metal co-doping on the quantum capacitance of supercapacitor electrodes based on mono- and multilayer graphene. Carbon.

[45] Madsen GKH, Singh DJ (2006). Boltztrap. A code for calculating band-structure dependent quantities. Comput. Phys. Commun..

[46] Cohen, M. L., Chelikowsky, J. R. & Louie, S. G. *Quantum Theory of Real Materials, Volume SECS 0348 of Kluwer International Series in Engineering and Computer Science* (Kluwer, 1996).

[47] Ziman, J. M. *Electrons and Phonons: The Theory of Transport Phenomena in Solids. Oxford Classic Texts in the Physical Sciences* (Clarendon Press and Oxford University Press, 2001).

[48] Hurd, C. M. *The Hall Effect in Metals and Alloys. The International Cryogenics Monograph Series* (Plenum Press, 1972).

[49] Khazaei M, Arai M, Sasaki T, Estili M, Sakka Y (2014). Two-dimensional molybdenum carbides: Potential thermoelectric materials of the mxene family. Phys. Chem. Chem. Phys..

[50] Hull, R. *et al.**Thermoelectrics* Vol. 45 (Springer, 2001).

[51] Khandy SA, Islam I, Gupta DC, Khenata R, Laref A (2019). Lattice dynamics, mechanical stability and electronic structure of fe-based heusler semiconductors. Sci. Rep..

[52] Yang GM, Zhang HZ, Fan XF, Zheng WT (2015). Density functional theory calculations for the quantum capacitance performance of graphene-based electrode material. J. Phys. Chem. C.

[53] Wang M, Chen L, Zhou J, Xu L, Li Xi, Li L, Li X (2019). First-principles calculation of quantum capacitance of metals doped graphenes and nitrogen/metals co-doped graphenes: Designing strategies for supercapacitor electrodes. J. Mater. Sci..

[54] Rani R, Sharma M, Bharti A, Sharma R (2023). Giant quantum capacitance and rashba splitting in tellurene bilayer derivatives. Phys. E Low Dimens. Syst. Nanostruct..

[55] Zhou Q, Wang L, Ju W, Yong Y, Dong Z, Chi S, Yao J (2023). Exploring of the quantum capacitance of mos2/graphene heterostructures for supercapacitor electrodes. FlatChem.

